# Acute and long-term toxicity in primary hypofractionated external photon radiation therapy in patients with localized prostate cancer

**DOI:** 10.1007/s00345-023-04714-3

**Published:** 2024-01-20

**Authors:** Wolfgang Lilleby, Amar Kishan, Hans Geinitz

**Affiliations:** 1 Department of Oncology, Oslo University Hospital, Oslo, Norway; 2 Department of Radiation Oncology, University of California, Los Angeles, Los Angeles, CA USA; 3 Department of Radiation Oncology, Hospital of the Barmherzigen Schwestern, Ordensklinikum, Linz, Austria

**Keywords:** Prostate cancer, Toxicity, Hypofractionation

## Abstract

**Background:**

Compelling evidence exists for the iso-effectiveness and safety of moderate hypofractionated radiotherapy (Hypo-RT) schedules [1, 2]. However, international guidelines are not congruent regarding recommendation of ultrahypofractionated radiotherapy (UHF-RT) to all risk groups.

**Methods:**

The current review gives an overview of clinically relevant toxicity extracted from major randomized controlled trials (RCT) trials comparing conventional to hypofractionated regimes in the primary setting of external photon radiation. Functional impairments are reported by using physician-rated and patient-reported scores using validated questionnaires.

**Results:**

The uncertain radiobiology of the urethra/bladder when applying extreme hypofractionation may have contributed to worse acute urinary toxicity score in the Scandinavian UHF-RT and worse subacute toxicity in PACE-B. The observed trend of increased acute GI toxicity in several moderate Hypo-RT trials and one UHF-RT trial, the Scandinavian Hypo-RT PC trial, could be associated to the different planning margins and radiation dose schedules.

**Conclusion:**

Nevertheless, Hypo-RT has gained ground for patients with localized PCa and further improvements may be achieved by inclusion of genetically assessed radiation sensitivity. Several RCTs in Hypo-RT have shown non-inferior outcome and well-tolerated treatment toxicity by physician-rated scores. In the future, we suggest that toxicity should be measured by patient-reported outcome (PRO) using comparable questionnaires.

## Introdution

Recent randomized controlled  trials (RCTs) have shown that hypofractionated radiotherapy (Hypo-RT) can be an effective treatment option for localized prostate cancer (PCa) [1–3]. Shorter treatment schedules achieved by moderate Hypo-RT or even more ultra-fractionated schedules (UHF-RT) compared to conventional RT (convRT) are of interest [4–6]. Hypo-RT regimes lead to decrease of number of fractions and number of treatment days, a factor associated with increased patient`s convenience, however, this has to be balanced with possible increased urinary and/or bowel toxicity.

Acute- and long-term toxicity can negatively impact patients’ quality of life (QOL) and may require additional medical intervention. Importantly, with a post-RT 10-year overall survival of >75% even in high-risk patients, such typical adverse events can greatly impact survivorship for patients with otherwise highly curable disease [6]. Given the high cure rate and longevity in general, relevant long-term toxicity contributes substantially in clinical decision-making process [7]. Considerable evidence supports the assumption that PCa has a relatively low alpha/beta ratio between 1.5 and 3.1 Gy for tumor versus 3 to 5 Gy for late-reacting tissue. These dose relationships of normal tissue complication probability and tumor control has been derived by well-conducted studies [8, 9]

In this review, we will comprehensively assess the acute- and long-term toxicity profiles of Hypo-RT/UHF-RT, beginning with a brief discussion of validated questionnaires for physician- and patient-rated outcomes. A detailed overview or meta-analysis of the use of these modalities as monotherapies for PCa are beyond the scope of this review.

## Toxicity outcome measures

### Physician-reported questionnaires

The current published RCTs in the field of hypofractionated radiation therapy in localized PCa used both physician and patient-reported outcomes (PROs) addressing QOL. Several comparable instruments have gained access to clinical research. The Radiation Therapy Oncology Group (RTOG) and the European Organization for Research and Treatment Group (EORTC) developed scoring criteria for acute and late radiation morbidity grading reactions after severity from 0 to 5 [10]. As reported by Trotti et al., the NCI Common Toxicity Criteria system (CTC) was the platform for the later developed CTCAE grading system. The CTCAE divides severity of adverse events into 5 grades [11].

### Patient-reported questionnaires

Patient-reported outcomes (PROs) aims to measure “any report of the status of a patient’s health condition that comes directly from the patient, without interpretation of the patient’s response by a clinician or anyone else” [12]. The goal is to evaluate the patient’s own views on their well-being, functioning, symptoms and experiences with treatment.

Usually (and traditionally), patients adverse events are reported by the examining physician in a follow-up setting such as outpatient clinics. However, patients tend to report more frequent and severe symptoms than the physician [13]. Generally, there is scientific consensus that subjective adverse events are ideally assessed using PROs rather than physician-based reports. The use of PROs should be increasingly used also in clinical trials by comparable instruments [14]. One excellent tool is the validated EPIC-50 questionnaire which has been abbreviated into two separate questionnaires, EPIC-26 and EPIC-16. EPIC-26 is shorter than EPIC-50 and does not examine sexual activity and sexual desire [15]. EPIC-26 is a brief, valid and reliable subjective measure of QOL among prostate cancer patients. It contains summary scores for the following domains: urinary incontinence, urinary irritation, bowel bother, sexual function and hormonal function. The shortest of the questionnaires, EPIC-16, excludes sexual function.

#### EORTC QLQ-C30

Another validated tool in PRO measurement outcomes is the EORTC Quality of Life Questionnaire (EORTC QLQ) and its 30-item core instrument (EORTQLQ-C30) measuring impairment grouped in 5 functional domains (physical, role, cognitive, emotional, and social functioning) [16]. The EORTC QLQ-PR25 is a disease-specific module with four domains focusing on sexual activity, urinary symptoms, bowel symptoms and treatment-related symptoms during the past 1 and 4 weeks [17].

#### PCSS

The Prostate Cancer Symptom Scale (PCSS) questionnaire addresses urinary, intestinal and sexual function on a scale from 0 (“no problem/very good function) to 10 (“many problems/very bad function”) [18].

#### SF-36 and SF-12

The above PROs questionnaires are often combined with generic QoL instruments. The Short Form-36 (SF-36) was developed in 1992 which was shortened to the more commonly used SF 12 item questionnaire in 1996 and later SF-12 [19]. The SF health surveys are the most widely used PRO tools in the world. All three variations of the SF survey measure the same eight health domains. The scores of these are further divided into two overall groups: [physical composite score (PCS, which consists of: physical functioning, role-physical, bodily pain, general health) and mental composite score (MCS, which consists of: vitality, social functioning, role-emotional and mental health). A score of 50 is usually set as the normal level in the general population [20].

### Radiation technology

The observed number of RCTs using Hypo-RT schedules in the last 2 decades evolved by rapid advances in modern radiotherapy techniques. Improvements in patient setup, control of organ movements by gating, fiducial markers, high-precision radiotherapy techniques IMRT/VMAT, stereotactic radiation (SBRT) and progress in radiological imaging have considerably contributed to exploration of Hypo-RT and UHF-RT in the localized PCa setting. These developments where doses were safely applied to the shape of the tumor have made it possible to increase radiation dose widening the therapeutic window.

### Monotherapy with moderate Hypo-RT

The PROFIT trial, a RCT phase 3 study with a median follow-up (FU) of 6 years, reported PROs (EPIC50 and SF-12) and physician-rated toxicity (RTOG toxicity score) (Table 1) in low-to-intermediate risk patients treated with moderate Hypo-RT (60 Gy in 20 fractions) versus conventional fractionation (78 Gy in 39 fractions) [2]. In the PROFIT trial, the use of IMRT technique was encouraged but 3D-CRT permitted and daily image guidance (IGRT) mandatory. Toxicity data were available for all patients for acute worst events during the first 14 days and for late worst events from 6 months onward. Physician-rated data were obtained from more than 90% of the patients at FU and cumulative toxicity is summarized in Table 1. GU toxicities were similar in both treatment arms. In the acute period, only 4% of patients in both arms had grade ≥ 3 GU toxicity; in the late period, 3.0% of patients in the convRT arm and 2.1% in the HypoRT arm experienced grade ≥ 3 toxicity. For GI toxicities, the proportion of patients with acute grade ≥ 3 toxicity was low in both arms. Late grade ≥ 3 toxicity was not significantly different between groups, but a trend toward higher levels in the standard arm (*P* = .10) was observed (Table 2).

**Table 1:** Acute and late organ-at risk toxicity Grade 2 or more

Study	Year	Number of patients	Dose Gy	ADT mo	acute GI (%) convRT/Hypo-RT	late GI (%) convRT/Hypo-RT	acute GU(%) convRT/Hypo-RT	late GU (%)convRT/Hypo-RT	FU mo
Hypo-RT									
PROFIT^a^	2017	598/608	78/60	None	10.5/16.7	13.9/8.9	31/30.9	22/22.2	60
RTOG0415a	2016	534/545	73.8/70	None	10.3/10.7	14.0/22.4	27.1 / 27	22.8/29.7	70
CHHiP^a;b^	2016	715/720/713	74/60/57	3–6	25/38/38	13.7/11.9/11.3	46/49/46	9.1/11/6.6	62
	2021	349/381/393				5.4/7.6/5.3			
		341/377/382						6.8/9.3/7.8	36
Hypro-RT^a;c^	2015	391/403	78/64.6/36*	31/42**					
	2016	387/395				17.7/21.9		39/41.3	
UHF-RT									
Hypo-RT-PC^a;b;c^	2019	578/569	78/42.7none 6/9	None	6/9	10/10	23/28	17/18	60
	2021	132/120						33/28	
		129/129				33/28			
PACE-B^a^	2019	432/415	78(62)/36.25	None	12/10		26.8/20.2		24
	2022	430/414				8.1/7.8		10.6/18.3	

a = physician-assessed toxicity; b = patient-reported toxicity; c = patient-reported bother incidence; mo = months; *significant; **ADT given in 66% of patients

**Table 2:** Acute and late organ-at risk toxicity Grade 3 or more

Study	Year	Number of patients	Dose Gy	ADT mo	acute GI (%) convRT/Hypo-RT	late GI (%) convRT/Hypo-RT	acute GU(%) convRT/Hypo-RT	late GU (%)convRT/Hypo-RT	FU mo
Hypo-RT									
PROFIT^a^	2016	598/608	78/60	None	0.7/0.7	2.7/1.5	4/3.9	3/2	60
RTOG 0415^a^	2016	534/545	73.8/70	None	< 1/ < 1	2.4/4.1	2.42/3	2/3	70
CHHiP^a;b^	2016	715/720/713	74/60/57	3–6	< 1/ < 1/ < 1	2/3/4	0/0/0	3/6/3	62
Hypro-RT^a;c^	2016	387/395	78/64.6	36	31/42	19/20	5/6	1/19	36
UHF-RT									
Hypo-RT-PC^a;b;c^	2019	578/569	78/42.7	None	0/1	1/ < 1	2 / 6	5 / 4	60
PACE-B^a^	2022	430/414	78(62)/36.25	None	1/ < 1	1/0	< 1/ < 1	< 1/ < 1	24

a = physician-assessed toxicity; b = patient-reported toxicity; c = patient-reported bother incidence; mo = months; N/A = not available; **ADT given in 66% of patients

A significant increase in acute grade ≥ 2 toxicity occurred in the Hypo-RT (*P* = .003); conversely, for late grade ≥ 2 toxicity, a significant increase occurred in the convRT arm (*P* = .006). A more detailed patient-reported functional symptoms and/or Qol analysis between treatment groups has not been published yet.

The RTOG 0415 evaluated treatment-induced toxicity in 1079 patients [3] treated with 70 Gy in 28 fractions versus 73.8 Gy in 41 fractions. The trial included only low-risk patients and ADT was not given in this non-inferior study design. They used the EPIC-50 questionnaire for PROs in the GI/GU domains and CTCAE version 3 for physician-reported toxicity (table1). At 24 months, the compliance rate was 61% with EPIC. The clinician-reported late adverse events compliance rate was 98%. Late Grade 2 for GI and GU toxicity were statistically significant increased in patients who were treated with Hypo-RT (p=0.005 resp. p=0.009). In their report, Lee et al. found no clinically relevant differences for the measured dimensions between treatment arms at any time point and for QoL. The authors concluded that Hypo-RT can be regarded as safe in treating low risk patients consistent with the findings by other moderate Hypo-RTs.

Of interest, in a sub-study, the authors analysed dosimetry and found an association between “hotspots” to rectal volume (D5%) as a relevant factor to long-term Grade 2 or more GI toxicity [21].

The CHHiP trial compared 60 Gy in 20 fractions against 57 Gy in 19 fractions and conventional fractionation 74 Gy in 39 fractions [1]. It included mainly intermediate risk patients (73%) but also high-risk patients (12%) assessed by NCCN risk stratification. The study included 1957 and data from 1141 patients (59%) were available at 6 year FU [22]. In the CHHiP trial, PROs instruments were changed in 2009 (protocol amendment March 2009) using the EPIC-50 and EPIC-26 questionnaires and HRQoL by SF-12 afterwards. Acute PROs were similar in the Hypo-RT arm compared to conventional or 19 fraction arm only. However, the RTOG grade 2 or worse for bladder toxicity was similar between the 74 Gy group and the 60 Gy group (p=0.34). General QoL were similar between schedules at 5 year (Table 1). The primary long-term QoL endpoint was bowel bother assessed by EPIC. There was some evidence for less sexual bother in the Hypo-RT schedules compared with the convRT schedules (p=0.009 for 60 Gy compared to 74 Gy). Meanwhile, they found no statistically significant differences for the overall bother scores and general QoL [22].

Of note, in the CHHiP trial, all patients were treated with IMRT but only ca 30% of all study patients received image guided RT.

Brand et al. analysed the fraction size sensitivity of late genitourinary toxicity in the CHHiP trial [9]. Using the CHHiP data, they applied Lyman–Kutcher–Burman (LKB) formula as a mathematical model to predict toxicity [23].

They found a relevant impact of the alfa/beta ratio related to the clinician-reported bladder adverse events. However, the lower than expected derived alfa/beta values (0.2–3 Gy) could narrow the therapeutic window for UHF-RT and possibly increase the risk of late side effects.

The HYPRO-RT trial, the only RCT with a superior outcome design, published acute and long-term side effect in two separate publications [24]. The trial enrolled 820 patients to Hypo-RT or convRT. Acute toxicity was reported by RTOG-EORTC for a time period of 120 days. HYPRO-RT showed significant greater acute GI toxicity grade 2 or more in the experimental arm (p=0.0015, Table 1). Long-term toxicity was reported for 3 years FU and compliance rate was 95%. The cumulative incidence of GU grade 3 was 19% for Hypo-RT arm and 12.9% in convRT arm (Table 2). The high late cumulative toxicity scores may reflect the method to report the highest of the two scores obtained from patient or clinician assessment of GI/GU impairment. The observed increase in adverse late events may further be due to less advanced radiation technique and prolonged treatment time. Additionally, in the high-risk group treatment of seminal vesicles to prescribed dose, pelvic nodes to 50 Gy, and long-term hormonal treatment was correlated with higher rate of late genitourinary toxicity.

### UHF-RT

The HYPO-RT-PC study, for example, was designed to give equal late toxicity in the two treatment arms. The majority of patients were treated with Linac 3D-conformal radiotherapy. The Scandinavian Hypo-RT-PC trial reported their PROs at 6-year follow-up [25]. PROs were defined as secondary outcome measures (Table 1). They used the validated EORTCQLQ-C30 and the PCSS questionnaires for measuring clinical relevant GI/GU/sexual functioning and bother as well as QoL. 158 (76%) of 233 patients in the convRT arm and 146 (66%) of 220 patients in the Hypo-RT arm completed the survey at 6 years (median FU 48 months; IQR 25-72). Acute mean scores for overall bowel bother were lower in the Hypo-RT group at the end of RT, with urinary scores significantly worse 3 months later. At 5-year FU, there were no differences in the PROs summarized domains for urinary, bowel, and sexual symptoms for bother and function and for QoL among Hypo-RT and convRT treated patients

PACE-B trial patients were treated with Cyberknife or Linac IMRT/VMAT. This phase 3 study used five fractions of UHF-RT and accepted convRT (31% of the patients) and moderate Hypo-RT (69% of the patients) in the control arm (Table 1) [4]. PACE-B included a per protocol pre-specified subanalysis for the co-primary endpoints of physician-rated GU/GI toxicity done by RTOG at baseline and follow-ups [4, 26]. It also contained the CTCAE version 3 grading as secondary endpoint. PROs were assessed at baseline and FU by EPIC-26 and symptom-specific questionnaires (Vaizey fecal incontinence score, International Prostate Symptom Score (IPSS), and International Index of Erectile Function 5-question score (IIEF-5). Baseline and 12 weeks at end of RT completion rates were at least 94% for RTOG and 88% for EPIC-26 in both groups. Patients treated with UHF-RT did have significantly worse acute CTCAE grade ≥2 GI toxicity exceeding baseline (15.2% vs. 8%, p=0.011), though this difference disappeared by 12 weeks.

The 2-year analysis included 430 patients in the Conv/Hypo-RT group and 414 in the UHF-RT group [26]. At 24 months, the physician-reported toxicity results were available for 91% (766 out of 844 patients, Table1). The prevalence of RTOG grade 2 or worse GU was 2% in the control RT group compared to 3% in the UHF-RT group at 2-year FU. The cumulative incidence of GU grade 2 or worse toxicity rates were 18.3% in the UHF-RT arm compared to 10.6% in the control RT group.

The prevalence of grade 2 or worse GU toxicity rates assessed by CTCAE criteria were 10.6% for Control RT and 18.3% for UHF-RT at 2 year FU. The cumulative incidence of GU grade 2 or worse toxicity rates were 19.8% in the control RT arm and 32.3% in the UHF-RT arm.

Prevalence of grade 2 GI toxicity was low, with no significant differences between groups at 2 years (according to RTOG criteria: 3% convRT v 2% in the UHF-RT Table 1). Follow-up for PACE-B is still short and survival data for PACE B has not been published. Kishan et al. reported outcome data using modern SBRT from 12 single-arm phase II studies [27].

In this pooled consortium analysis, the 7-year cumulative incidence of Grade 3 was 2.4% for GI toxicity and 0.4% for GI toxic events. Given the impact of tight planning margins on toxicity recently published data from the MIRAGE trial using MRI guidance may further reduce toxicity over time [28].

#### PACE A

When technical improvements lead to less treatment induced toxicity, the focus changes to sustain or regain of organ function. However, there is a paucity of data on this topic using PROs.

Filling this gap, PACE-A a superior trial randomized 123 men with localized PCa to either stereotactic UHF-RT or prostatectomy [29]. Co-primary endpoints were patient reported outcomes (PROs) of EPIC-26 questionnaire number of absorbent pads per day and EPIC bowel subdomain score at 2 years. Secondary endpoints included clinician-reported toxicity. The long-term toxicity was recently presented. At a median FU of 50 months, UHF-RT patients had significantly worse bowel subdomain score (mean (SD) 88.4 (12.7) vs 97.3 (5.5), *p* < 0.001). None of the patients in both groups reported grade 2 or worse bowel problems and 7/56 (15.6%) UHF-RT and 0/45 (0%) surgery patients reported grade 1–2 problems (*p* = 0.04, only one patient with moderate problems in the UHF-RT-arm). At 2 years, 4.5% and 46.8% of the patients in the UHF-RT and surgery group, respectively, reported use of at least one pad/day for urinary incontinence. PACE-A contributes with clinically important findings relevant to informed decision making.

### Boost combined with EBRT

Exploring further the concept of focal dose escalation, the first long-term findings of the FLAME study has been published [30]. FLAME is a phase III multicenter RCT that randomized mostly high-risk patients to conventional 77Gy in 35 fractions with or without an integrated boost up (2.7 Gy) to 95 Gy to multiparametric MRI-defined (macroscopic) tumor within the prostate. ADT (at least 6 months or more) was permitted and used in 65% of the patients. The study incorporated EORTC-QLQ-C30 and CTCAE version 3.0 as tools for grading toxicity. The two groups had similar acute and cumulative late GI and GU toxicities (cumulative incidence GU 23% vs. 28% for the boost arm; GI 12% vs. 13%, respectively). There was no statistically significant difference in HRQoL between both treatment arms.

In a similar trial, termed hypo-FLAME, patients with intermediate- and high-risk PCa were treated with 35Gy stereotactic UHF-RT in 5 fractions to the whole prostate with and integrated boost up to 50Gy to the MRI-defined tumors also demonstrated favorable GU/GI toxicity profiles [31].

The prostate-only radiotherapy (POP-RT) trial compared PROs in men with very high-risk prostate cancer to patients receiving prophylactic whole-pelvic nodal RT [32]. They used 68 Gy with 25 fractions to the prostate and 50 Gy with 25 fractions to whole-pelvic nodes. The acute GU/GI toxicities graded with RTOG score were similar in both groups, late bowel and bladder toxicities showed no differences for grade 2 or more GI toxicity (8.2% v 4.5%, *P* = 0.28), but cumulative grade 2 or more late GU toxicity was significantly higher with whole-pelvic RT (20.0% v 8.9%, *P* = 0.02).

### Future perspectives

In the published trials, physician-rated 5-year late grade ≥2 GU toxicity rates range from 12 to 15% [33]. Most of the moderate-to-severe toxicity after RT will develop in the first 2 years [34]. However, the cumulative incidence does insidiously increase over time with 10-year rates of 17–20% reported on clinical trials [35] and population registry data confirming an increased risk of significant toxicity compared to the general population.

The EAU guidelines recommend treatment with UHF-RT preferably in trials, given the uncertainty of long-term toxicity. In this regard, the long-term urinary toxicity of the PACE A and B trial is eagerly awaited.

However, many patients with PCa are elderly, with a substantial chance of developing lower urinary tract symptoms over a 5-year period in the absence of PCa treatment. Physicians tend to underestimate organ bother impairments after curatively intended treatment, therefore, it is important to integrate PROs by validated questionnaires when introducing new treatment regimes [14].

One approach to detect patients` with increased risk of inborne radiosensitivity comes from considerable data suggesting that genomic factors may be important in determining clinical radiosensitivity. Several studies suggest that germline single nucleotide polymorphisms (SNPs) in multiple genes may be associated with GU toxicity after radiotherapy, however, those reported have only modest accuracy for predicting toxicity [36]. While the genomic basis of radiosensitivity in most patients remains unknown, emerging data suggest an important role for microRNAs (miRNAs)—small, non-coding RNA elements miRNAs are global regulators of stress response pathways, including the local and systemic response to radiation [37].

A phase II validation trial for this potential biomarker has completed accrual (NCT04624256). Even if the absolute overall toxicity rates are similar for patients treated with Hypo-RT or convRT, such panels may help to identify patients who are more likely to experience significant toxicity after one treatment regimen or the other.

In this line, the results of the ongoing NRG GU009, implementing a genetic classifier, are eagerly anticipated. Together, advances in biomarker development, genetic signatures and pattern recognition of images may allow patients and physicians to obtain a roadmap of risk adapted, individualized treatment options.

## Conclusion

RCTs with Hypo-RT showed pronounced acute toxicity but long-term adverse effects did not differ from convRT. PROs analysis of QoL reassured the long-term safety of hypofractionated radiotherapy in men with localized PCa.

## Data Availability

Not applicable.
